# Perspective on human diagnostic and national reference laboratory preparedness for zoonotic influenza in Europe

**DOI:** 10.2807/1560-7917.ES.2026.31.27.2500918

**Published:** 2026-07-09

**Authors:** Zandra C Felix Garza, Lance D Presser, Dirk Eggink, Angeliki Melidou, Eeva K Broberg, Adam Meijer

**Affiliations:** 1 Centre for Infectious Diseases Research, Diagnostics and Laboratory Surveillance, National Institute for Public Health and the Environment (RIVM), Bilthoven, the Netherlands; 2 European Centre for Disease Prevention and Control (ECDC), Stockholm, Sweden

**Keywords:** Influenza, Zoonotic, External Quality Assessment, EQA, Nucleic Acid Amplification Test, NAAT, Sequencing, Diagnostic Laboratory, Reference Laboratory

## Abstract

Zoonotic influenza A viruses (zIAV) originating from avian and swine reservoirs present a serious concern for public health. Since the emergence of zIAV A(H5N1) in 1996, the virus has spread globally, impacting wild birds, poultry, wild and domestic mammals, including cattle and pigs, and sporadically infecting humans. Challenges persist in detecting and characterising zIAV, particularly at the human–animal interface. Recent external quality assessments conducted in the Netherlands and at European level have evaluated the capacity of human clinical diagnostic and national reference laboratories to detect, subtype and characterise potential zIAV. Based on these results, we reflect on the status and challenges of methods used for identifying cases of zIAV infection in Europe. While all laboratories are largely successful in generic detection of influenza A virus, subtyping is not widely used in clinical diagnostic laboratories. For national reference laboratories, subtyping specifically of swine-origin viruses remains challenging, often requiring sequencing for accurate identification. Although sequencing offers greater potential for characterising zIAV, training in the appropriate use of bioinformatics tools is needed. Raising awareness among healthcare professionals to document animal exposure in patient disease histories is also critical, as early suspicion of zoonotic infections is needed to direct laboratory testing, including subtyping.

## Background

Zoonotic influenza A viruses (zIAV), particularly those emerging from avian and swine reservoirs, are a serious concern for public health. Since the emergence of H5N1 avian influenza A virus in 1996, highly pathogenic for poultry, and a first human infection detected in 1997 [1], outbreaks have occurred in wild birds and poultry, and various genetic clades have emerged [2]. Avian influenza viruses that are high or low pathogenic for poultry can both infect humans [3]. Until 2014, highly pathogenic avian influenza (HPAI) A(H5N1) was mostly observed in South-East Asia and Egypt [4]. Since 2021, HPAI A(H5N1) has affected millions of wild birds and poultry in the European Union/European Economic Area (EU/EEA) and worldwide and has led to infections in various wildlife and domestic mammalian species [2,5], including recent outbreaks among cattle in the United States (US) [6] and transmission to pigs [5,7]. Pigs are susceptible to human, avian and swine influenza A viruses (IAV) [8], which increases the risk of coinfections that may produce reassorted influenza viruses with pandemic potential, successfully transmissible between humans. Until now, noted human infections with avian or swine IAV remain sporadic and are mostly caused by direct contact with infected animals [9,10]. Nevertheless, the potential threat to human health is high, which is why the European Commission has ranked the monitoring of zoonotic influenza as a top priority in their One Health approach, recognising the connection between human, animal and environmental health in pathogen detection [11]. Guidelines have been published for testing and detection of zIAV infections in Europe [12,13], and the international health regulations (IHR) have been amended to strengthen surveillance and accelerate laboratory response time to potential public health emergencies of international concern [14]. Also, the European Centre for Disease Prevention and Control (ECDC) continuously performs public health emergency preparedness assessments [15]. In addition, European surveillance and outbreak reporting tools such as the Early Warning and Response System (EWRS) [16] and EpiPulse [17] have been implemented to facilitate rapid sharing of information. However, guidelines, assessments and tools alone are not sufficient, their success relies on the early detection, surveillance, reporting and response efforts at a country level.

In the last years, influenza virus detection and genomic surveillance have improved substantially, in part due to technological developments implemented in response to the COVID-19 pandemic [18]. In addition, following a One Health approach, several European countries have implemented active and passive monitoring programmes for people at high-risk of exposure and infection with zIAV, such as farm workers, veterinarians and people involved in culling activities [19,20]. The structure of human surveillance and diagnostic pipelines may vary per country [21] but often involves five layers for symptomatic cases (Figure): 

**Figure:**
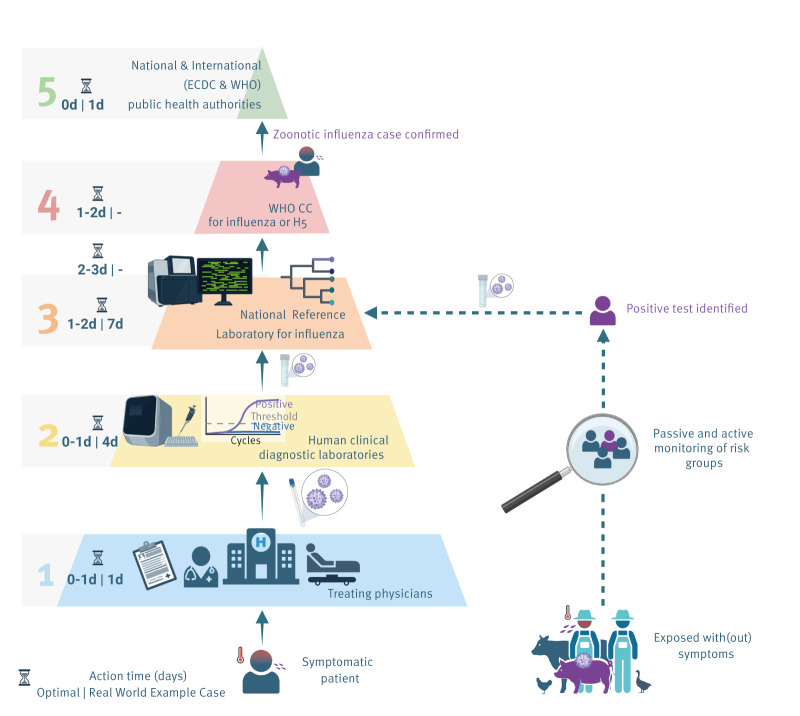
General structure of surveillance and diagnostics for influenza virus in Europe in the context of identifying a human infection with animal influenza virus

ECDC: European Centre for Disease Prevention and Control: WHO CC: World Health Organization Collaborating Centre.

Timely detection and characterisation of zoonotic influenza A cases involves close collaboration and communication between five layers. The structure of the surveillance programmes and the tasks of the first three layers may vary between countries. However, in general, a symptomatic patient might have been exposed to animals infected by influenza A virus, but exposure is often only known in specific programmes for passive and active monitoring of exposed individuals or when asked in Layer 1 as part of the patient’s disease history review. In Layer 2, specimens are tested for influenza A virus. In Layer 3, influenza A virus-positive specimens are thoroughly examined to determine the host origin, (sub)type and genetic characteristics of the influenza virus. However, the National Reference Laboratories may not always have the capacity to perform a full virus characterisation independently and may require support from the WHO CC for influenza and/or WHO Reference laboratory for H5 (Layer 4) to confirm a suspected case of zoonotic influenza. Once a zoonotic influenza case is confirmed, the case is reported to the national and international public health authorities in Layer 5. The optimal total response time is 4-9 days if confirmation by WHO CC is needed. If results of the National Reference Laboratory are considered conclusive and virus is submitted to WHO CC for reference purposes only, the total optimal response time is reduced to 2–4 days. In practice, however, there are several factors that may impact the response time as illustrated by the real-world example of an A(H1N2)v swine influenza case [10] with a response time of 13 days. The figure has been created in BioRender; Felix Garza, Z. (2026). https://BioRender.com/o3mp60h.

1. Initial case assessment by treating physicians or municipal health services,
2. Testing for influenza viruses at human clinical diagnostic laboratories (hCDL), needed for patient management,
3. Further analysis of influenza virus-positive specimens suspected to be of animal origin to determine the host origin, (sub)type and genetic characteristics of the influenza virus; done by the national reference laboratory for human influenza (NRL) in a given country, which is often a World Health Organization (WHO) National Influenza Centre,
4. Confirmation of a zoonotic influenza case with the support of the WHO Collaborating Centres (CC) for Reference and Research on Influenza and/or WHO H5 Reference Laboratories,
5. Reporting of the confirmed zIAV case to national and international public health authorities, such as the national ministry of health, the ECDC (through EWRS, EpiPulse) and the WHO (through IHR).

However, the link of an individual to potentially infected animals is not always clear, particularly when contact was indirect, the disease was mild or asymptomatic in the infected animals, or several animal exposures were reported. These ambiguous links make surveillance and diagnostics challenging, especially when the infected person presents mild symptoms and may not consult a physician [10].

These challenges emphasise the need for accurate clinical diagnosis of zIAV infected cases, including robust laboratory testing at hCDL, and also for solid protocols at NRLs for zIAV detection and characterisation, including the specific animal subtype of the virus, e.g. swine or avian. Thus, a logical question is: ‘Can European hCDL and NRLs detect zIAV, especially of avian and swine origin, with the tests and protocols currently in use?’. Recently, two independent external quality assessments (EQAs) addressed this question and examined the extent to which avian and swine IAV can be detected, subtyped and characterised by hCDL in the Netherlands [22] and NRLs in the European Union/European Economic Area (EU/EEA), Western Balkan countries, and Türkiye [23]. Here, we reflect on the status of molecular diagnostics and characterisation of potential zIAV in Europe, based on the results from these recent EQAs, and highlight key aspects to address in our path to stronger pandemic preparedness, which may be applicable globally.

## External quality assessments on zoonotic influenza

The two recent independent EQAs on zoonotic influenza, i.e. the Dutch EQA for hCDL in the Netherlands [22] and the European EQA for NRLs within the ECDC European External Influenza Quality Assessment Programme (EEIQAP) [23], used testing panels with similar avian, swine and human origin IAV, including common subtypes and varying viral loads. Details of these EQAs can be found in Supplementary Table S1 and the respective EQA reports [22,23]. The Dutch hCDL that participated in the Dutch zIAV EQA mainly used widely available commercial nucleic acid amplification tests (NAAT), many of which are probably also used by other hCDL in Europe [24]. The NRLs that participated in the European EQA used mostly in-house laboratory developed tests (LDT). 

## Molecular diagnostics

The results from both EQAs showed that the Dutch hCDL and the European NRLs can successfully detect IAV in specimens containing potentially zoonotic viruses of avian and swine origin using either commercial or LDT NAAT, with some variations in detection sensitivity. Although, to the best of our knowledge, the Dutch EQA is the first in Europe to focus on the simultaneous detection of various avian and swine IAVs in hCDL, others have recently examined whether hCDL in diverse European countries can detect avian IAV H5N1 clade 2.3.4.4b using similar commercial NAAT [24]. Their conclusions are in agreement with those of the Dutch EQA.

Beyond the generic detection of IAV however, the zoonotic influenza EQAs showed that subtyping is not widely implemented in Dutch hCDL, but is available at NRLs. This finding is not surprising given the current role of hCDL and NRLs in the layers of surveillance and diagnostics (Figure) and the fact that knowing the virus subtype is not needed for patient treatment of IAV infection in the absence of animal exposure history. For the same reason the number of commercial NAAT that include at least subtyping for seasonal H1pdm09 and H3 is limited [22]. This observation is also reflected in European surveillance reports where the vast majority of non-sentinel influenza A virus detections are not subtyped [25]. NAAT-based subtyping at European NRLs is mostly focused on haemagglutinin (H)-subtyping, e.g. H1, H3, H5 and H7, and is limited for neuraminidase (N). The European EQA showed that only 50% of the participating NRLs also performed NAAT-based N-subtyping, despite its relevance for the detection of reassortants and confirmation of H-subtyping results. Subtyping human seasonal and avian (H5 and H7) IAV was successfully performed at almost all NRLs using their readily available NAAT. Importantly though, subtyping of swine-origin viruses remains challenging and often requires sequencing for accurate identification. The latter is due to assay limitations when using NAAT-based subtyping, mainly because of cross-reactivity between the human seasonal and swine IAV subtypes in LDT and commercial NAAT [22,23]. This also reflects the challenge of subtyping an unexpected emerging zoonotic virus for which NAAT is not readily available, rendering the specimen unsubtypable and thus relying on referral for sequencing.

## Sequencing-based characterisation

Considering the limitations of NAAT, sequencing-based subtyping and characterisation of human and animal IAV is crucial, especially at NRLs, for the detection of zIAV infections and for a rapid local response. Sequencing of the haemagglutinin and neuraminidase genome segments of IAV should be the minimum for adequate identification of animal IAV infection. However, whole genome sequencing is preferred as it provides a complete picture of the virus’s genetic makeup, enabling identification of reassortment and of markers for mammalian adaptation and reduced antiviral susceptibility. In the European EQA, 70% of the participating NRLs reported sequencing results. In addition, among the laboratories that did report their whole genome sequencing results, only 57% provided the correct H- and N-subtype and the likely host species for all influenza viruses in the panel. This success rate may seem low, but review of the reported raw data from the European EQA [23] suggests that the main challenge was in reporting the interpretation of the findings rather than the sequencing result itself. Furthermore, based on the uploaded sequences, sequencing data allowed for the identification of specific markers for antiviral susceptibility and mammalian adaptation, but several laboratories probably lacked the tools or knowledge to identify them. This indicates a need for strengthening sequencing capabilities (implementation of sequencing, sequence analysis and data interpretation) at the NRLs and developing bioinformatics training pathways focused on the use and interpretation of bioinformatics tools for virus characterisation, genotyping and marker identification.

## Challenges in early detection of zoonotic influenza A viruses

The outcome of the Dutch and European EQAs made evident that there is a good level of laboratory preparedness in Europe to identify and characterise potential zIAV when suspicion of animal IAV infection is clear. The main challenge lies in the detection of zIAV when the case is not derived from active or passive monitoring of individuals exposed to HPAI virus. A clear example is the case of a Dutch patient whose swine influenza virus infection was detected by coincidence, although there was a clear exposure to swine [10]. Particularly challenging are the cases where the individual may not think of seeking medical assistance or the treating physician may not think of asking the patient about exposure to animals potentially infected with influenza virus. This emphasises the need for awareness campaigns aimed at the general population and treating physicians. Individuals should have enough information to conclude early on that they need to seek medical assistance. Treating physicians, on the other hand, should be aware of the need to thoroughly inventorise and document a patient’s animal exposure. There are various guidelines to aid healthcare professionals in recognising possible human cases of zIAV infection [26,27], but they might not always be aware of the relevance of documenting a patient’s direct or indirect contact with livestock and wild animals.

## Strengthening pandemic preparedness

The risk of animal IAV to human health calls for continuous evaluation and improvement of our pandemic preparedness systems. External quality assessments such as those discussed here are crucial for this process and provide essential information for the participating laboratories and public health authorities. In this context, the two recent zIAV EQAs point out key aspects that should be addressed on our path to more robust laboratory preparedness.

### Sensitivity of influenza A virus nucleic acid amplification-based detection

Generic detection of IAV using NAAT was excellent among participating Dutch hCDL and NRLs in their respective EQAs [22,23], although variation was observed in the detection sensitivity of the methods used. This suggests an area of opportunity for European hCDL and NRLs to review the performance of their NAAT for generic detection of type A influenza viruses and adjust their methods accordingly, particularly if LDT are used. In addition, hCDL and NRLs must be mindful of the challenges that the recent In Vitro Diagnostic Regulation entails for both commercial kits and LDTs, particularly in an outbreak or pandemic setting [28]. It is crucial that test manufacturers, of both commercial kits and LDTs, perform a regular monitoring of their test performance and update them accordingly. In the case of LDTs, an annual in silico evaluation of primers and probes should be performed in order to update them when needed, especially when mutations are thought to affect their performance. Frequent in silico evaluation of commercial tests, especially for those that include H-subtyping [22], are the responsibility of the manufacturers because they do not release information on used primers and probes.

### Nucleic acid amplification test-based subtyping

Many different H- and N-subtyping NAAT are in use, as documented in the reports from the European EQA [23] and ECDC’s survey on laboratory capacity for molecular diagnosis and characterisation of zIAV in human specimens [29]. The NAAT-based subtyping at the European NRLs is adequate for haemagglutinin subtyping of human seasonal IAV and avian H5 and H7 IAV but not always successful in the H-subtyping of swine IAV. Capacity for NAAT-based H- and N- subtyping should be enhanced in the NRLs for the main zIAV subtypes, especially for those of swine origin and their distinction from human subtypes.

### Whole genome sequencing of influenza viruses and genetic characterisation

As a preparedness action for potential outbreaks, NRLs should prioritise increasing their capacity and capabilities to perform and interpret whole genome sequencing of influenza viruses. Sequencing of at least HA and NA genome segments of type A influenza viruses must be considered a critical capacity and expertise of NRLs. In addition, because of the constant technological advances in this area, bioinformatics training should be revised and reinforced. Efforts should focus on generating consensus sequences, assigning clade and subclades, identifying the genotype for influenza virus A(H5N1), and identifying amino acid markers for reduced antiviral susceptibility and mammalian adaptation. We also support continuous development of publicly available tools like INSAFLU [30] and of continuously updated mutation databases like FluMut [31]. In addition, participation in sequencing [23] and bioinformatics [32] external quality assessments is crucial for NRLs to identify challenging aspects in their protocols and analysis workflows based on the current quality metrics.

### Implementation of external quality assessment-like studies at the national level

The NRLs, or the equivalent national bodies, could also consider conducting EQA-like studies with local hCDL to assess readiness for zIAV detection using their routine influenza diagnostic tests; the Dutch EQA [22] and another recent EQA that focused on detection of influenza virus A(H5N1) clade 2.3.4.4b [24] can be used as a guidance. This type of study could also aid in identifying potential performance issues inherent to commercial tests and contribute to the improvement of these.

### Awareness campaigns aimed at treating physicians

Continuous efforts should be set to raising awareness among physicians to include exposure to animals potentially infected with IAV in the patient’s disease history review, especially of severe hospitalised influenza A cases, as also advised in ECDC surveillance guidance documents [12,13,33]. One example on how to implement this is through promoting literacy among healthcare professionals on zoonoses [34] and on the current epidemiological situation in their area.

## Conclusion

There is a good level of preparedness in Europe to identify and characterise potential zIAV when suspicion of animal IAV infection is clear. Nevertheless, various actions are needed to strengthen our preparedness level for detecting potential zIAV when the link to an influenza virus-infected animal is less evident.

## Data Availability

After termination of the ECDC AURORAE project in 2026, anonymised data on the ECDC zoonotic influenza EQA are available on reasonable request from ECDC. Data on the Dutch zoonotic influenza EQA are available on reasonable request for which inquiries can be sent to the corresponding author.

## Supplementary Data

175000 Supplement
