# Supplementary material for: Perspective on human diagnostic and national reference laboratory preparedness for zoonotic influenza in Europe
Source: Euro Surveill. 2026 Jul 9;31(27):2500918. doi: 10.2807/1560-7917.ES.2026.31.27.2500918 (PMC13354935; doi:10.2807/1560-7917.ES.2026.31.27.2500918)
Supplement: Supplement [file 25-00918_MEIJER_Supplement.pdf]

Supplementary material

This supplementary material is hosted by Eurosurveillance as supporting information alongside the article Perspective on human diagnostic and national reference laboratory preparedness for zoonotic influenza in Europe, on behalf of the authors, who remain responsible for the accuracy and appropriateness of the content. The same standards for ethics, copyright, attributions and permissions as for the article apply. Supplements are not edited by Eurosurveillance and the journal is not responsible for the maintenance of any links or email addresses provided therein.

Supplementary Table S1. Panel compositions of the Dutch EQA for human clinical diagnostic laboratories and the European EQA for national reference laboratories for human influenza in EU/EEA, Western Balkan countries and the Republic of Türkiye (full details in references [1, 2]).

		**National EQA for human clinical diagnostic laboratories in the Netherlands**	**European EQA for national reference laboratories for human influenza in EU/EEA, Western Balkan countries and the Republic of Türkiye**
**Total number of specimens**		10	15
**Specimens per host**	**Avian**	4	9
	**Human**	2	2
	**Swine**	3	3
	**Negative control**	1	1
**Subtypes per host**	**Avian**	• H5N1 clade 2.3.4.4b from 1 genotype (AB) at 2 dilutions (started with sample at Ct 26.5 RIVM influenza A Matrix RT-qPCR and further diluted 1:16.6 at Ct 30.1; both expected to test positive).<br>• H5N6 clade 2.3.4.4b<br>• H7N2[a] clade 1.1	• H5N1 clade 2.3.4.4b, from 3 different genotypes (AB, BB and C), genotype AB comprised a 10-fold serial dilution series from the second dilution (started with sample at Ct 18.2 RIVM influenza A Matrix RT-qPCR and further diluted 1:10,000, 1:100,000, 1:1,000,000 and 1:10,000,000; last two dilutions expected to test negative).<br>• H5N8 clade 2.3.4.4c<br>• H7N2[a] clade 1.1
	**Human**	• H1N1pdm09[a] clade 6B.1A.5a.2a.1 subclade C.1.1<br>• H3N2[a] 3C.2a1b.2a.2a.1 subclade G.1.1	• H1N1pdm09[a] clade 6B.1A.5a.2a.1 subclade C.1.1<br>• H3N2[a] 3C.2a1b.2a.2a.1 subclade G.1.1
	**Swine**	• H1N1 1C.2.1 (from human case [3])<br>• H1N2[a] 1C.2.2 (from human case [3])<br>• H3N2 1970.1 (Netherlands, 1999)	• H1N1 1C.2.2 (from human case [3])<br>• H1N2[a] 1C.2.2 (from human case [3])<br>• H3N2 1970.1 (France, 2015)

a. This virus was identical in both EQAs.

References

1. Zoomer S, Goderski G, van den Brink S, Presser LD, Felix Garza ZC, Vuong O, van der Vries E, Houben M, Fouchier RAM, Eggink D, Meijer A. Zoonotic influenza preparedness: Dutch medical labs efficiently detect animal influenza A viruses - External Quality Assessment, 2023 J Clin Virol. 2026;183:105927. https://doi.org/10.1016/j.jcv.2026.105927. Epub ahead of print. PMID: 41734493.
2. European Centre for Disease Prevention and Control. External quality assessment for molecular detection, subtyping and characterisation of potentially zoonotic type A influenza viruses, 2024. Stockholm: ECDC. Available from: https://www.ecdc.europa.eu/sites/default/files/documents/20250520_AURORAE_zoonotic_EQA.pdf, 2025 (accessed 2026 Mar 26).
3. Eggink D, Kroneman A, Dingemans J, Goderski G, van den Brink S, Bagheri M, Lexmond P, Pronk M, van der Vries E, Germeraad E, Brandwagt D, Houben M, Hooiveld M, van der Giessen J, van Gageldonk-Lafeber R, Fouchier R, Meijer A. Human infections with Eurasian avian-like swine influenza virus detected by coincidence via routine respiratory surveillance systems, the Netherlands, 2020 to 2023. Euro Surveill. 2025;30(19):2400662. https://doi.org/10.2807/1560-7917.ES.2025.30.19.2400662. PMID: 40376819.
